# Cardiovascular System Changes and Related Risk Factors in Acromegaly Patients: A Case-Control Study

**DOI:** 10.1155/2015/573643

**Published:** 2015-10-27

**Authors:** Xiaopeng Guo, Lu Gao, Shuo Zhang, Yilin Li, Yue Wu, Ligang Fang, Kan Deng, Yong Yao, Wei Lian, Renzhi Wang, Bing Xing

**Affiliations:** ^1^Department of Neurosurgery, Peking Union Medical College Hospital, No. 1 Shuaifuyuan, Beijing 100730, China; ^2^Peking Union Medical College, No. 5 Dongdansantiao, Beijing 100730, China; ^3^Department of Clinical Laboratory, Peking Union Medical College Hospital, No. 1 Shuaifuyuan, Beijing 100730, China; ^4^Department of Cardiology, Peking Union Medical College Hospital, No. 1 Shuaifuyuan, Beijing 100730, China

## Abstract

*Background*. Cardiovascular complications are known to be the main determinants of reduced life expectancy and decreased quality of life in acromegaly patients. Our study aimed to provide insight into the cardiovascular changes that occur in acromegaly patients and to investigate the correlative risk factors. *Methods*. A total of 108 patients definitively diagnosed with acromegaly and 108 controls matched for age and gender were recruited into study and control groups, respectively. Standard echocardiography was performed on all of the participants, and data were collected and analyzed. *Results*. All acromegaly patients presented with structural cardiac changes, including a larger heart cavity, thicker myocardial walls, and increased great vessel diameters compared with the control group. Additionally, the acromegaly patients presented with reduced diastolic function. Aging and increased body mass index (BMI) were correlated with myocardial hypertrophy and diastolic dysfunction; a longer disease duration was correlated with larger great vessel diameters. *Conclusions*. Ageing and increased BMI are independent risk factors for acromegalic cardiomyopathy, and a long disease duration results in the expansion of great vessels. Increased efforts should be made to diagnose acromegaly at an early stage and to advise acromegaly patients to maintain a healthy weight.

## 1. Introduction

Acromegaly is characterized by excessive growth hormone (GH) secretion and is primarily caused by a GH-secreting pituitary adenoma, which stimulates the growth of various tissues and impairs the structures and functioning of the heart and great vessels [1–3]. Cardiovascular disease is known to be the most important complication of acromegaly, accounting for the increased morbidity and decreased life expectancy of these patients [2, 4]. In the absence of other known cardiac diseases, cardiovascular disease in acromegaly is characterized by acromegalic cardiomyopathy, which was first defined in 1895 [5]. The most common feature of acromegalic cardiomyopathy is concentric biventricular hypertrophy [6], which consists of both structural and functional abnormalities [7], including concentric biventricular hypertrophy and diastolic dysfunction [8, 9], which cause the progression of the final heart failure with a preserved ejection fraction. Although risk analysis studies are common in the field, some controversial viewpoints remain in the literature regarding the risk factors for acromegaly. Left ventricular hypertrophy has been mainly considered to be correlated with GH levels, whereas exercise tolerance response and systolic function are mainly correlated with disease duration and the presence of diabetes and hypertension [2, 4, 6, 8, 10]. Ambiguity remains regarding the risk factors for the expansion of the great vessels. In this study, we evaluated the clinical features and echocardiography results of 108 patients with acromegaly and 108 gender- and age-matched individuals. We aimed to detect the morphological and functional cardiovascular changes and to investigate the underestimated risk factors for enlarged heart cavity, thickened myocardial walls, and increased great vessel diameters in acromegaly patients in China.

## 2. Materials and Methods

### 2.1. Study Population

The Department of Neurosurgery of Peking Union Medical College Hospital (PUMCH) admitted a total of 146 acromegaly patients from January to December in 2013. The study group consisted of 108 patients who were recruited strictly according to the inclusion criteria, which were as follows: (1) pathologically diagnosed with acromegaly; (2) of any age and of either gender presenting with elevated GH and insulin-like growth factor 1 (IGF-1) levels and normal levels of other hormones, including thyroxin, hormones of hypothalamic-pituitary-adrenal axis, and gonadal hormones; (3) no history of surgery, radiotherapy, or medical treatment of somatostatin analogs before hospital admission; (4) no history of treatment with hormonal replacement for pituitary deficit; and (5) no known cardiac disorders. The control cases, who were gender- and age-matched with the study group, were selected from the Department of Physical Examination Center of PUMCH in 2013. Patients in the control group had normal pituitary function, no previous endocrine diseases, and no known cardiac disorders.

The diagnosis of acromegaly was based on the following criteria [11, 12]: (1) typical symptoms of acromegaly, for example, enlarged hand/foot and thickening of the soft tissue; (2) lack of suppression of GH to below 1.0 ng/mL after oral administration of 75 g glucose; (3) a high level of fasting GH (>2.5 ng/mL); and (4) a high level of serum IGF-1 controlled for age and gender. After documentation of their disease information, all patients underwent hormone assays, MRI evaluation, and echocardiography after admission.

### 2.2. Ethics Statement

The present study was approved by the Institutional Ethics Committee of PUMCH, Chinese Academy of Medical Sciences, and the clinical investigation was conducted according to the principles expressed in the Declaration of Helsinki. Each recruited subject provided written consent before entering the study.

### 2.3. Hormone Assays and Laboratory Diagnosis

The serum GH level was measured using a chemiluminescence assay (L2KGRH2, Siemens Healthcare Diagnostics Products Ltd., Glyn Rhonwy, Llanberis, Gwynedd LL55 4EL, UK) and an IMMULITE 2000 analyzer; in all of the acromegaly patients, the assay was performed the day after hospital admission after an eight-hour fasting period and administration of a 75 g oral glucose tolerance test. The serum IGF-1 level was measured using a chemiluminescence assay (L2KGFZ, Siemens Healthcare Diagnostics Products Ltd., Glyn Rhonwy, Llanberis, Gwynedd LL55 4EL, UK) with the same analyzer as that used for measuring GH, and the levels in the acromegaly patients were compared with those of control individuals of the same age and gender. The levels of other pituitary hormones, including adrenocorticotropic hormone, cortisol, prolactin, follicle-stimulating hormone, luteinizing hormone, estrogen 2, progestin, testosterone, free thyroxine, free triiodothyronine, thyroxine, triiodothyronine, thyroid-stimulating hormone, and parathyroid hormone, were determined by electrochemiluminescence assays (Roche Diagnostics GmbH, Mannheim, Germany) with an ADVIA Centaur XP (Siemens) analyzer. All serum samples were collected at 6 a.m. after an eight-hour fasting period.

### 2.4. Sellar MR Images

All MRI studies were performed with the same magnetic resonance machine (3.0 Tesla, Discovery MR750, GE). All of the following images were obtained for valid patient diagnosis: T1- or T2-weighted coronal sequence, T1-weighted sagittal sequence, and gadolinium-enhanced T1 coronal sequence. All of the MRI studies were conducted by the same doctor (BH) and analyzed by the same neuroradiologist (FF), who had more than 5 years and 20 years of experience, respectively, in sellar MRI interpretation. The neuroradiologist was strictly blinded to the participant status and study aim. The following characteristics of adenoma were recorded: the maximum diameter and lateral invasion of the cavernous sinus and the vertical extension (suprasellar and inferior) and signals in the T1, T2 and gadolinium-enhanced images.

### 2.5. Echocardiographic Examination

All of the patients were scanned by the same physician (LF) at the Department of Cardiology using a Vivid E9 ultrasound scanner (GE, Norway) with a 2.5 MHz transducer before surgery. The cardiologic physician was also strictly blinded to the participant status and study aim. Standard echocardiograms were recorded to obtain morphologic and functional parameters. Color-coded tissue Doppler images were also obtained to determine strain.

All of the echocardiography procedures were performed by the same practitioner at the Department of Cardiology. The following data were recorded: interventricular septum (IVS), posterior wall of left atrium (PW), the E (early wave of the mitral flow)/A (atrial contraction wave of the mitral flow) ratio, left ventricular ejection fraction (LVEF), left ventricular fractional shortening (LVFS), left ventricle end-diastolic diameter (LVEDD), left ventricle end-systolic diameter (LVESD), left atrium longitudinal dimension (LALD), right ventricle longitudinal dimension (RVLD), aortic root dimension (AORD), ascending aorta (AA), main pulmonary artery (MPA), and postcava diameters.

### 2.6. Statistical Analysis

Statistical analyses were performed using SPSS, version 17.0 (SPSS Inc., IBM, USA). The results are expressed as percentages or mean ± SD. The Kolmogorov-Smirnov test was used to evaluate the normality of the distribution of each parameter. Comparisons of categorical variables and proportions were analyzed using the Chi-squared test. The unpaired *t*-test was used to assess differences between the two groups in variables with normally distributed values, and the Mann-Whitney test was used to examine differences between groups in variables lacking normal distributions. Binary logistic regression was used to analyze eleven covariates between the cardiovascular disease and noncardiovascular disease groups. A *p* value of <0.05 was considered statistically significant.

## 3. Results

### 3.1. Study Population

The demographic information, clinical characteristics, and GH and IGF-1 levels of the two groups are presented in Table 1. In the study group, MRI findings showed sellar masses in all of the patients, which appeared hypointense on T1-weighted MRI and had decreased contrast enhancement compared to the surrounding normal tissue. Macroadenomas (90.7%, 98/108, 2.5 ± 2.2 cm) were more often present than microadenomas (9.3%, 10/108, 0.8 ± 0.1 cm) in the study group. The average maximum diameter of the macroadenomas was 2.5 cm, and 28.6% (28/98) of them invaded cavernous sinus. In contrast, the microadenomas had an average maximum diameter of 0.8 cm and showed no cavernous sinus invasion.

Sex and age were matched between the study and control groups, and no differences in BMI, smoking, hypertension, or dyslipidemia were found. More acromegaly patients than control subjects had diabetes mellitus (*p* < 0.001). The disease duration of acromegaly (defined as the time since the first symptoms connected with acromegaly to a definitive diagnosis) was 77.8 ± 63.0 months (range: 1–360 months). The nadir GH level was 21.4 ± 39.3 ng/mL (mean ± SD, range: 1.1–214.0 ng/mL), the fasting GH level was 27.4 ± 44.6 ng/mL (range: 2.7–276.0 ng/mL), and the IGF-1 level was 802.7 ± 337.5 ng/mL (range: 144.0–1591.0 ng/mL) in the acromegaly group.

### 3.2. Cardiovascular Morphological and Functional Parameters

We divided the echocardiography parameters into the following categories: heart cavity expansion, myocardial hypertrophy, great vessel expansion, diastolic function, and systolic function. The morphological and functional cardiovascular parameters of both groups are presented in Table 2.

With regard to heart cavity expansion, LVEDD, LVESD, LALD, and RVLD were all larger in the study group than in the control group (49.9 ± 5.0 mm versus 46.6 ± 4.1 mm, 31.0 ± 4.3 mm versus 28.9 ± 3.3 mm, 36.0 ± 4.9 mm versus 32.1 ± 3.9 mm, and 22.3 ± 3.9 mm versus 21.0 ± 5.2 mm, resp., all *p* < 0.05).

With regard to myocardial hypertrophy, IVS and PW were 8.7 ± 1.6 mm and 8.5 ± 1.3 mm, respectively, in the study group and 7.4 ± 1.1 mm and 7.5 ± 1.0 mm, respectively, in the control group. The differences in these values were significant between the two groups.

With regard to great vessel expansion, AA (31.6 ± 3.9 mm), AORD (31.8 ± 3.3 mm), and MPA (22.2 ± 2.6 mm) were larger in the acromegaly patients than in the control individuals (AA (29.5 ± 3.7 mm), AORD (29.9 ± 3.3 mm), and MPA (21.1 ± 2.6 mm), all *p* < 0.05), whereas the postcava diameter did not differ between the two groups (*p* = 0.36).

To assess diastolic function decline, we evaluated the E/A ratio, which is the most meaningful indicator of the diastolic functioning of the heart. The acromegaly patients exhibited a decreased E/A ratio (1.2 ± 0.3) compared with the healthy individuals (1.3 ± 0.3) (*p* < 0.001).

With regard to systolic function decline, no differences in the LV ejection fraction or LV fractional shortening were noted between the study and control groups (67.3 ± 5.6 mm versus 68.1 ± 5.2 mm, *p* = 0.25; 37.6 ± 4.4 mm versus 38.2 ± 4.1 mm, *p* = 0.29).

### 3.3. Risk Factors and Determinants of Cardiovascular Changes

We divided the acromegaly patients (study group members) into different subgroups based on condition: with/without myocardial hypertrophy, with/without heart cavity expansion, with/without great vessel expansion, and with/without diastolic function decline. The data for each condition and differences between the patients with and without each condition are presented in Table 3.

In general, no differences were found in the prevalence of the four above-mentioned conditions with regard to gender, smoking, or IGF-1 level (*p* > 0.05 in all comparisons). In contrast, age, BMI, diabetes mellitus, dyslipidemia, and disease duration were found to be positively related to myocardial hypertrophy. In addition, diastolic function decline shared the same potential risk factors, and hypertension, fasting GH, and nadir GH levels were also correlated with diastolic function decline in acromegaly. Disease duration was related to great vessel expansion.

The results of regression analyses for the acromegaly patients are shown in Table 4. Age (*p* = 0.002, OR = 1.14) and BMI (*p* = 0.03, OR = 1.24) were the most important predictors of myocardial hypertrophy for these patients. The elderly (*p* = 0.002, OR = 1.14) or those with an increased BMI (*p* = 0.02, OR = 1.26) were also susceptible to diastolic dysfunction. In addition, great vessel expansion was significantly correlated with disease duration (*p* = 0.006, OR = 1.05).

## 4. Discussion

Although acromegaly is widely considered to be a rare disease, it is not trivial with regard to its morbidity and mortality [2, 4]. Acromegaly is caused by GH hypersecretion in adults and has a detrimental effect on the cardiovascular system; specifically, cardiomyopathy is a well-known consequence of this condition [6, 10, 13]. Myers et al. performed echocardiography on 11 cats and reached a similar conclusion [14]. The common cardiovascular changes that occur in acromegaly include heart cavity expansion, myocardial hypertrophy, left ventricular systolic and diastolic dysfunction, enlarged great vessel diameters, arrhythmia, and increased prevalence of valve diseases [1, 15–17]. These cardiovascular complications are major determinants of the shortened life expectancy of acromegaly patients. Patients with acromegaly have an approximately 30% increased mortality rate compared with healthy individuals, and cardiovascular disease is the cause of death in 60% of these patients [18].

In this study, a higher proportion of diabetes was observed in the acromegaly patients compared with the control group. Heart cavity expansion, myocardial hypertrophy, diastolic dysfunction, and expansion of the great vessel diameters were also more often present in the acromegaly patients. Aging and increased BMI were positively correlated with myocardial hypertrophy and diastolic dysfunction. In addition, a longer disease duration was correlated with expanded great vessel diameters.

Myocardial hypertrophy is common in acromegaly, occurring in approximately 90% of acromegaly patients with hypertension and long disease durations. Colao et al. found that left ventricular hypertrophy is more common in patients over 50 years of age than in younger patients [9, 19, 20]. Our study found that age, BMI, diabetes mellitus, and dyslipidemia were significantly higher in the acromegaly patients than in the controls; however, hypertension was not found to correlate with myocardial hypertrophy.

The decline in left ventricular diastolic function is thought to develop during the early stages of acromegaly. Moreover, systolic dysfunction appears before the progressive impairment of systolic performance, with the latter leading to heart failure [9, 21, 22]. Our results demonstrated that diastolic function was lower in acromegaly patients and that the decline in diastolic function in acromegaly (20.4%, 22/108) was positively related to increased age and BMI.

No difference in systolic function was found between the two groups. This indicated that our patients were diagnosed at an early stage of heart impairment before diastolic dysfunction. This observation leads us to conclude that cardiac systolic function decline should not be emphasized over diastolic dysfunction, as has been the case.

Minimal data are available regarding the expansion of great vessel diameters in acromegaly patients [1, 16, 17]. The great vessel diameters comprise the inner diameters of the aortic root, ascending aortic root, main pulmonary artery root, and postcava. To the best of our knowledge, only three studies have addressed great vessel diameters in acromegaly, with most of them focusing on the aortic root diameter [1]. We noted that the ascending artery, aortic root dimension, and main pulmonary artery, but not the postcava, differed between the acromegaly patients and controls, indicating that vascular changes in the acromegaly patients only occurred in the arterial system. We examined the postcava diameter in the acromegaly patients, and our results provide the first evidence that it is not a determinant of great vessel expansion. Unexpectedly, comparisons between the two groups indicated that all of the acromegaly patients had the same likelihood of developing heart cavity expansion.

Although it has been shown that GH and IGF-1 have indispensable roles in the development of cardiovascular complications in acromegaly patients, we did not find a statistically significant difference in either hormone between acromegaly patients and the controls. In fact, it is the long-term exposure to GH and IGF-1 rather than the high levels of these hormones that determined the course of acromegalic cardiomyopathy. This finding, which indicates that cardiovascular changes in acromegaly are not aggravated by high levels of GH or IGF-1, may be an important milestone for research on acromegaly-related diseases.

Transsphenoidal pituitary surgery, the gold-standard treatment for pituitary adenomas, including acromegaly, has become a commonly performed neurosurgical procedure in recent years. Nonetheless, this surgery presents particular challenges to anesthesiologists because of the perioperative cardiopulmonary dysfunctions in acromegaly. Acromegaly patients are more likely to develop acute heart failure and arrhythmia during the perioperative period [23, 24]. Therefore, increased attention should be focused on the anesthesia risks associated with acromegaly and the early detection of cardiovascular system dysfunction in acromegaly patients; in particular, the use of echocardiography will help to decrease perioperative anesthesia risks.

Our study has some limitations. The subjects were only selected from a single medical center over the course of one year, and the number of study subjects was not sufficient. We only compared different risk factors according to the echocardiography findings and did not follow up by recording their cardiac outcomes after surgery. Multicenter studies should be conducted in the future, and future meta-analyses are recommended. A follow-up of these patients is ongoing, which will allow us to analyze cardiac structural and functional parameters after surgery and to determine whether the related cardiovascular changes are reversible.

## 5. Conclusion

Acromegaly patients have a high risk of developing cardiovascular complications, especially myocardial hypertrophy, artery diameter expansion, and diastolic dysfunction, which lead to increased risks of operative anesthesia and postoperative morbidity and mortality. Based on the results of our study, age, BMI, and disease duration should receive more attention in acromegaly patients than they have in the past. Increased efforts should be made to diagnose acromegaly at an early stage and to advise acromegaly patients to maintain a healthy weight to protect their cardiovascular system from constant damage.

## Figures and Tables

**Table 1:** Demographic, clinical, and endocrine characteristics.

	ACM (*n* = 108)	Control (*n* = 108)	*p* value
Male, *n* (%)	44 (41)	44 (41)	>0.99
Age, years	41.2 ± 12.7	41.2 ± 13.5	0.79
BMI, kg/m^2^	27.4 ± 5.7	26.2 ± 4.0	0.11
Smoking, *n* (%)	46 (43)	53 (49)	0.34
HT, *n* (%)	30 (28)	41 (38)	0.11
DM, *n* (%)	59 (55)	25 (23)	<0.001
Dyslipidemia, *n* (%)	15 (14)	17 (16)	0.70
Nadir GH, ng/mL	21.4 ± 39.3	—	—
Fasting GH, ng/mL	27.4 ± 44.6	—	—
IGF-1, ng/mL	802.7 ± 337.5	—	—
DD, month	77.8 ± 63.0	—	—

The data are presented as mean ± SD. ACM = acromegaly; BMI = body mass index; HT = hypertension; DM = diabetes mellitus; IGF-1 = insulin-like growth factor 1; and DD = disease duration.

**Table 2:** Morphological and functional cardiovascular parameters.

	ACM (*n* = 108)	Control (*n* = 108)	*p* value
Heart cavity			
LVEDD, mm	49.9 ± 5.0	46.6 ± 4.1	<0.001
LVESD, mm	31.0 ± 4.3	28.9 ± 3.3	<0.001
LALD, mm	36.0 ± 4.9	32.1 ± 3.9	<0.001
RVLD, mm	22.3 ± 3.9	21.0 ± 5.2	0.04
Myocardial thickness			
IVS, mm	8.7 ± 1.6	7.4 ± 1.1	<0.001
PW, mm	8.5 ± 1.3	7.5 ± 1.0	<0.001
Great vessel diameter			
AA, mm	31.6 ± 3.9	29.5 ± 3.7	<0.001
AORD, mm	31.8 ± 3.3	29.9 ± 3.3	<0.001
MPA, mm	22.2 ± 2.6	21.1 ± 2.6	0.003
Postcava, mm	14.3 ± 2.7	13.9 ± 3.1	0.36
Diastolic function			
E/A	1.2 ± 0.3	1.3 ± 0.3	<0.001
Systolic function			
LV ejection fraction, %	67.3 ± 5.6	68.1 ± 5.2	0.25
LV fractional shortening, %	37.6 ± 4.4	38.2 ± 4.1	0.29

ACM = acromegaly; LVEDD = left ventricle end-diastolic diameter; LVESD = left ventricle end-systolic diameter; LALD = left atrium longitudinal dimension; RVLD = right ventricle longitudinal dimension; IVS = interventricular septum; PW = posterior wall; AA = ascending aorta; AORD = aortic root dimension; MPA = main pulmonary artery; E = early wave of the mitral flow; A = atrial contraction wave of the mitral flow; and LV = left ventricle.

**Table 3:** Relationship between echocardiography and potential risk factors.

	HCE+ (*n* = 30)	HCE− (*n* = 78)	*p*	MCH+ (*n* = 19)	MCH− (*n* = 89)	*p*	GVE+ (*n* = 8)	GVE− (*n* = 100)	*p*	DFD+ (*n* = 22)	DFD− (*n* = 86)	*p*
Male, *n*	16	28	0.10	6	38	0.37	4	40	0.86	7	37	0.34
Age, years	42.6 ± 12.8	40.7 ± 12.7	0.48	52.9 ± 7.4	38.7 ± 12.2	<0.001	46.4 ± 14.6	40.8 ± 12.6	0.24	53.1 ± 7.7	38.2 ± 12.0	<0.001
BMI, kg/m^2^	28.5 ± 4.4	27.0 ± 6.1	0.24	31.6 ± 9.4	26.6 ± 4.1	<0.001	29.5 ± 5.4	27.3 ± 5.7	0.30	31.7 ± 8.7	26.4 ± 4.0	<0.001
Smoking, *n*	17	29	0.07	10	36	0.33	4	42	0.95	12	34	0.20
HT, *n*	10	20	0.42	8	22	0.13	5	25	0.06	10	20	0.04
DM, *n*	15	44	0.55	15	44	0.02	5	54	0.92	17	42	0.02
Dyslipidemia, *n*	6	9	0.41	6	9	0.04	0	15	0.52	7	8	0.02
Nadir GH, ng/mL	29.8 ± 60.4	18.1 ± 27.2	0.31	11.4 ± 12.9	23.5 ± 42.7	0.23	38.9 ± 71.9	20.0 ± 35.8	0.49	8.7 ± 11.1	24.7 ± 43.2	0.003
Fasting GH, ng/mL	32.9 ± 64.5	25.3 ± 34.3	0.54	18.2 ± 26.2	29.4 ± 47.4	0.33	54.3 ± 92.3	25.2 ± 38.5	0.41	11.3 ± 11.8	31.5 ± 48.8	0.001
IGF-1, ng/mL	809.6 ± 403.3	800.0 ± 311.4	0.91	802.4 ± 278.0	802.7 ± 350.2	>0.99	940.8 ± 340.9	791.6 ± 336.5	0.23	753.5 ± 306.0	815.2 ± 345.6	0.45
DD, month	93.9 ± 58.2	71.6 ± 64.0	0.09	101.1 ± 77.9	72.8 ± 58.6	0.04	172.5 ± 50.1	75.8 ± 63.6	<0.001	108.1 ± 77.3	70.0 ± 56.7	0.002

HCE = heart chamber expansion; MCH = myocardial hypertrophy; DFD = diastolic function decline; GVE = great vessel expansion; HT = hypertension; DM = diabetes mellitus; BMI = body mass index; DD = disease duration; and IGF-1 = insulin-like growth factor.

**Table 4:** Determinants of changes in the cardiovascular system.

		HCE			MCH			GVE			DFD	
	OR	95% CI	*p*	OR	95% CI	*p*	OR	95% CI	*p*	OR	95% CI	*p*
Age, years	1.03	0.98–1.08	0.19	1.14	1.05–1.24	0.002	1.20	0.99–1.47	0.07	1.14	1.05–1.24	0.002
BMI, kg/m^2^	1.01	0.93–1.09	0.89	1.24	1.02–1.50	0.03	0.84	0.60–1.20	0.34	1.26	1.03–1.53	0.02
DD, month	1.00	0.99–1.01	0.27	1.00	0.99–1.01	0.73	1.05	1.02–1.09	0.006	1.00	0.99–1.02	0.39

HCE = heart chamber expansion; MCH = myocardial hypertrophy; DFD = diastolic function decline; GVE = great vessel expansion; BMI = body mass index; and DD = disease duration.
